# Measurement and conceptualization of male involvement in family planning: a bibliometric analysis of Africa-based studies

**DOI:** 10.1186/s40834-024-00293-9

**Published:** 2024-06-13

**Authors:** Tosin Olajide Oni, Rebaone Petlele, Olufunmilayo Olufunmilola Banjo, Akinrinola Bankole, Akanni Ibukun Akinyemi

**Affiliations:** 1 Department of Demography and Social Statistics, Obafemi Awolowo University, Ile-Ife, Nigeria; 2 Department of Demography and Population Studies, University of the Witwatersrand, Johannesburg, South Africa; 3 The Guttmacher Institute, New York, USA

**Keywords:** Family planning, Male involvement, Men, Africa, Measurements

## Abstract

**Background:**

Male involvement in Family Planning (FP) is an exercise of men’s sexual and reproductive health rights. However, the measurement of male involvement has been highly inconsistent and too discretional in FP studies. As a result, we used bibliometric tools to analyze the existing measures of male involvement in FP and recommend modifications for standard measures.

**Methods:**

Using developed search terms, we searched for research articles ever published on male involvement in FP from Scopus, Web of Science, and PubMed databases. The search results were filtered for studies that focused on Africa. A total of 152 research articles were selected after the screening, and bibliometric analysis was performed in R.

**Results:**

Results showed that 54% of the studies measured male involvement through approval for FP, while 46.7% measured it through the attitude of males to FP. About 31% measured male involvement through input in deciding FP method, while others measured it through inputs in the choice of FP service center (13.6%), attendance at FP clinic/service center (17.8%), and monetary provision for FP services/materials (12.4%). About 82.2% of the studies used primary data, though the majority (61.2%) obtained information on male involvement from women alone. Only about one in five studies (19.1%) got responses from males and females, with fewer focusing on males alone.

**Conclusion:**

Most studies have measured male involvement in FP through expressed or perceived approval for FP. However, these do not sufficiently capture male involvement and do not reflect women’s autonomy. Other more encompassing measures of male involvement, which would reflect the amount of intimacy among heterosexual partners, depict the extent of the exercise of person-centered rights, and encourage the collection of union-specific data, are recommended.

**Supplementary Information:**

The online version contains supplementary material available at 10.1186/s40834-024-00293-9.

## Background

Family planning (FP) plays a crucial role in improving sexual and reproductive health (World Health Organization [1]. FP entails using contraceptive methods to space births or limit the number of childbirths to the desired level [2]. Thus, FP is used interchangeably with contraceptive use. FP allows people to delay or space pregnancies, thereby making it a veritable tool for reducing risks of pregnancy complications associated with closely spaced pregnancies [3]. Health interventions have also relied on FP to reduce infant morbidity and mortality that may result from unplanned pregnancies, especially among adolescents at both ends of the reproductive age (15–49) [1]. The proper and consistent use of male and female condoms, which are FP methods, has also proven effective in protecting individuals from sexually transmitted infections such as gonorrhea and chlamydia [4]. Among HIV-discordant couples or sexual partners, condoms have been an effective tool for preventing the transmission of HIV [5]. Moreover, FP can help individuals and couples build financial security by allowing them to raise a family that they can adequately care for.

The interpersonal relationships inherent in sexual activity make the involvement of males and females in FP crucial to maximizing its benefits [6]. Traditionally, FP has been conceived as women’s affairs since females play the biological role of carrying pregnancies and bear a greater burden of childrearing [7]. However, with increasing levels of information and awareness of sexual and reproductive health rights [8], there has been a paradigm shift in the role of men in FP. Male involvement in FP is now recognized as an exercise of men’s sexual and reproductive health rights [9]. Through their involvement, men can benefit from FP counseling, improve their knowledge of contraceptive choices, and protect themselves from health risks [10]. Moreover, men’s knowledge and involvement are essential to support women’s FP use, given that women use most methods.

In Africa, male involvement in FP has peculiar significance because of the prevailing patriarchy that weaves men’s dominance into societal norms, favoring men as key decision-makers [11, 12]. Moreover, partner attitudes and beliefs about sexual and reproductive health impact women’s utilization of FP services, especially in settings where the uptake and consistent use of contraception, desired family size, and timing of pregnancies are controlled by men [6, 13, ]. For instance, women who use contraception without the knowledge or consent of their partners are more likely to discontinue use compared to those whose partners are involved in the decision to use a method [14]. As a result, men play essential roles in determining women’s uptake of FP and the continuity of FP use [15]. Also, men’s involvement in FP is vital for policy and programs that aim to advance the achievement of FP goals in Africa. To this end, numerous studies and FP interventions have recommended that male involvement be incorporated into FP programs [12, 16].

However, despite the widely acknowledged importance of male involvement in achieving FP goals [12, 17], there have been no standard metrics for measuring it. The measurements adopted have been highly inconsistent and too discretional. For instance, some studies measured male involvement as communication and discussion with male partners about contraception [18, 19]. Some conceptualized male involvement as men’s perceptions and attitudes towards family planning [20, 21], while some measured it as approving the use of FP [22, 23]. Another measure that has also been adopted is the use of male method of contraception, as well as men accompanying their partners to the clinic [10]. Studies rely on one or a combination of these activities to measure men’s involvement in family planning [18–21], thus drawing attention to the inconsistency in measurement.

Conceptualizing and measuring male involvement is crucial for maximizing men’s involvement in achieving FP goals. The lack of a standard measure is a methodological gap in empirical investigations [24]. Hence, there is a need to review the measures adopted in existing studies to analyze the extent of their adoption and evaluate their validity to recommend modifications where required. A veritable tool to achieve this is bibliometrics [25, 26]. Bibliometric analysis is a scientific computer-assisted statistical technique that helps review studies’ methodologies and metadata and their relationships by covering all the publications related to a defined topic or field [27]. The computing power of bibliometrics enables it to review many studies, and researchers have leveraged this power [25, 26]. Bibliometrics has been used to analyze childhood immunization research productivity [28] and COVID-19 research output in Africa [29]. In this study, we used Bibliometrics to analyze the measurements of male involvement in African FP studies.

## Methods

### Data source and search strategy

We searched for published articles within the title, abstract, and keyword query string of Scopus, Web of Science, and PubMed databases. We developed search terms by combining keywords using Boolean operators (AND, OR, NOT) and Boolean logic (TRUE, FALSE). The search terms used include “male involvement” OR “partner involvement” OR “men involvement” OR “husband involvement” OR “male participation” OR “partner participation” OR “men participation” OR “husband participation"’ AND ‘contraceptive OR “family planning” OR contraception’ (see appendix A for the complete query string).

### Eligibility and study selection

**Fig. 1:**
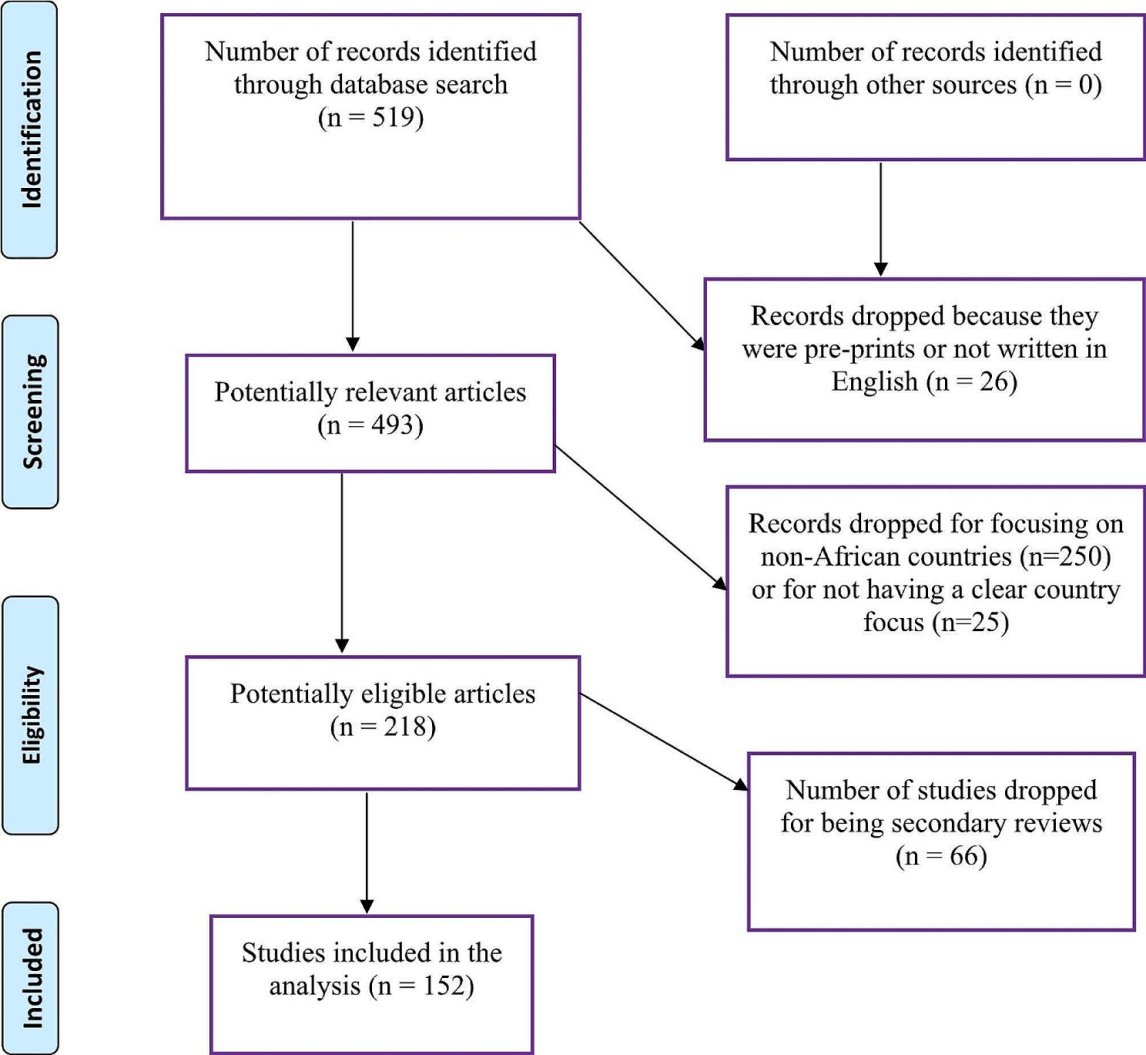
PRISMA Flow Diagram

A total of 519 search results were found. Four were pre-prints, and 22 were non-English, which were removed. The remaining 493 articles were filtered for African countries, leaving the search output with a total of 218 research articles as: Ethiopia (42), Nigeria (38), South Africa (26), Uganda (21), Kenya (18), Ghana (13), Tanzania (11), Malawi (9), Rwanda (8), Mozambique (5), Zimbabwe (4), Senegal (4), Zambia (3), Cameroon (3), the Democratic Republic of Congo (2), Congo (2), Botswana (2), Angola (2), Togo (1), Somalia (1), Sierra Leone (1), Egypt (1) and Burkina Faso (1). We manually checked the abstract and title of the remaining 218 articles to select studies that made a direct conclusion about male involvement either as the outcome on its own or as a factor (variable) influencing contraceptive practice or family planning. During the manual checks, 66 articles were removed because they were secondary reviews or did not measure male involvement in FP. Thus, we were left with 152 research articles. The screening flowchart of the research articles is illustrated in the PRISMA Flow Diagram shown in Fig. 1.

Table 1 presents an overview of the main information of the research documents reviewed in this study. The 152 research articles were written by 916 authors and published in 81 academic journals. Each paper had an average of 5 authors and about 17 citations. The annual growth rate in producing research articles focusing on male involvement in family planning in Africa is 7.2%.

### Data Analysis techniques

We carried out a descriptive analysis to show the basic characteristics of the selected research articles. Some characteristics are: (i) the time span, measured as the period range within which the selected articles were published; (ii) International co-authorship is measured as the proportion of research articles with at least one of the authors affiliated with an institution outside the country of study; and (iii) Document average age, measured as the average number of years since the articles were published, and annual growth rate – the percentage increase in the number of articles published within two consecutive years. A co-occurrence network analysis was conducted with VOSviewer to depict relations among the keywords used for database search (see Appendix B). We carried out a bibliometric analysis of the selected articles using Bibliometrix, which is an R package for mapping analysis of scientific studies [25, 26].

## Results

Table 1 shows that the studies analyzed were published between 1996 and 2023, with 916 authors at an average of 5 authors per research paper. The 152 analyzed papers were published in 81 academic journals, each with an average of 17 citations.

**Table 1:** Basic information of the analyzed studies

Variables	Values
Time span	1996–2023
Authors	916
Authors of single-authored docs	4
Co-authors per Doc	5.29
Sources (*academic journals*)	81
Documents	152
Document Average age	6.12
Average Citations per doc	16.65
International Co-authorship	53.37%
Annual growth rate	7.18%

**Fig. 2:**
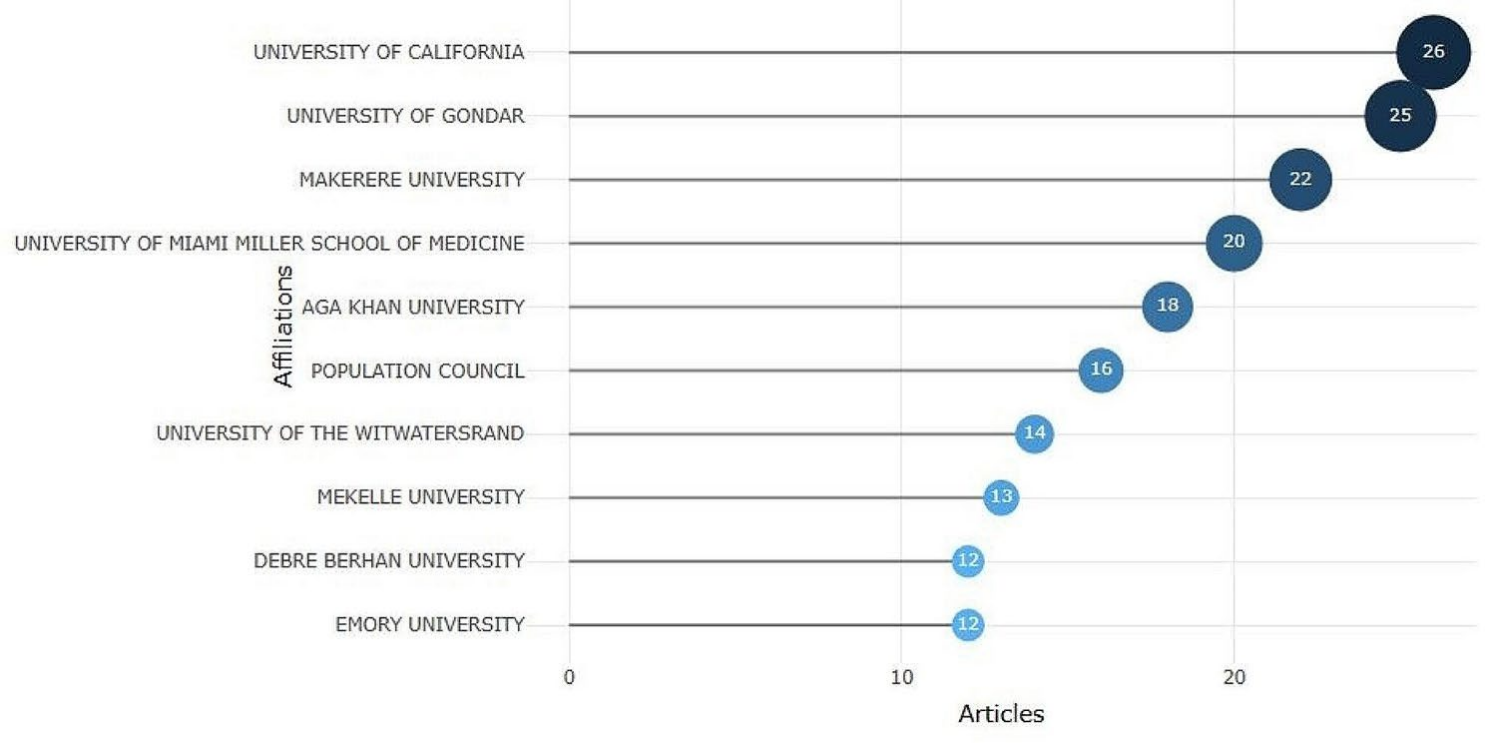
Most relevant affiliations

As presented in Fig. 2, results show that authors affiliated with the University of California, University of Gondar, and Makerere University had the top three volumes – 26, 25, and 22, respectively - of research articles that focused on male involvement in family planning in Africa.

**Fig. 3:**
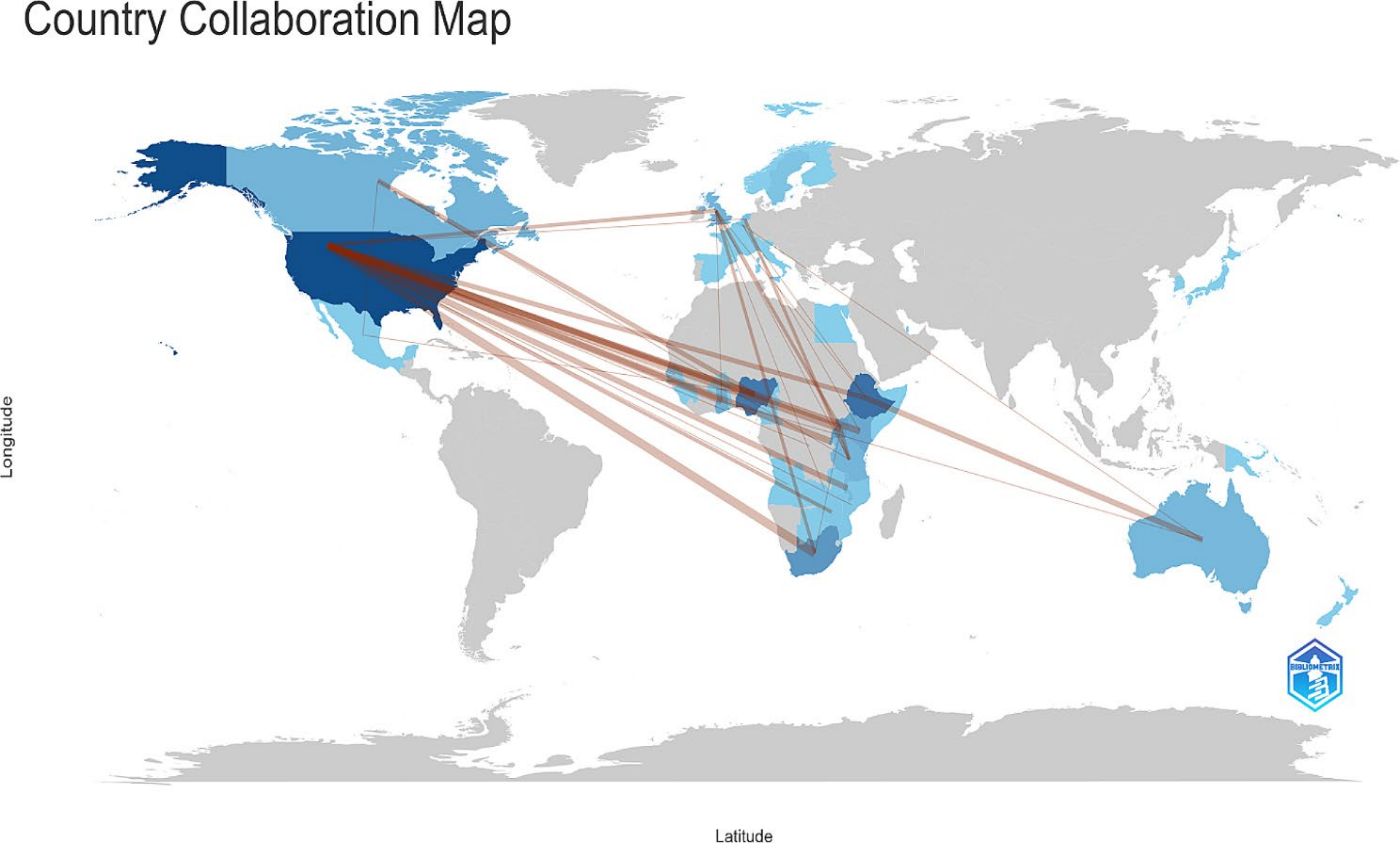
Collaboration Word Map

Figure 3 shows, using graded colours, the pattern of country collaboration among authors to research male involvement in family planning in Africa. As shown on the World Map, the country that collaborated most with African countries was the United States. The United Kingdom, Canada, and Australia followed this. The top three African countries collaborated with Nigeria, South Africa, and Ethiopia.

Table 2 shows how the authors of the reviewed studies have measured and conceptualized male involvement in FP in Africa. These are: (i) Perceived support or positive attitude of males towards FP. This was measured by asking whether or not men would support or would not go against the use of FP or contraceptives. e.g., *Would your husband support your use of FP or contraceptives?*; (ii) Expressed approval or consent of males for FP: This question asked whether or not men expressed support for using FP or contraceptives. e.g., *Did your husband/partner support your use of FP/contraceptives?*; (iii) Male involvement in the discussion about FP/contraceptive: This measure referred to whether or not the respondents and partner had engaged in any discussion about their use or intention to use FP; (iv) Male involvement in FP/contraceptive method choice: This measured whether men had any input in choosing an FP method being used or to be adopted; (v) Male involvement through monetary provision for FP service or material costs: This measured whether the male partner ever provided money to pay for the cost of FP materials or services; (vi) Self-use: This measured whether the male partner used any method of FP/contraception; (vii) Choice of FP service center: This measured whether the male partner was involved in deciding the choice of place/facility where FP services were accessed; and (viii) Attendance at FP clinic or service center: This measured whether husbands/male partners accompanied their partners or wives to the FP clinic/service center.

The results show that the commonest way (54%) through which authors measured male involvement in family planning was by asking if men approved of the use of family planning or contraception (see Table 2). Another way it was measured in 46.7% of the studies was by asking women if they thought or perceived that their partners would support their use of family planning. About 42% of the authors measured male involvement by asking if men engaged or participated in family planning discussions with their partners. About 28% measured male involvement through men’s use of contraceptive methods, while the least adopted measure was whether male partners made input or participated in deciding where to access family planning services.

**Table 2:** Measurement and conceptualisation of male involvement

How studies have measured and conceptualized men’s involvement in FP	The proportion of studies that adopted specific measurements (%)
Expressed approval or consent of males for FP	54.0
Perceived support or positive attitude of males towards FP	46.7
Male involvement in the discussion about FP/contraceptive	41.5
Male involvement in FP/contraceptive method choice	30.9
Self-use by male	28.3
The male attended or accompanied his partner to the family planning clinic/service center	17.8
Male involvement in the choice of place to access FP/contraceptive services	13.6
Male involvement through monetary provision for FP service or material costs	12.4

**Fig. 4:**
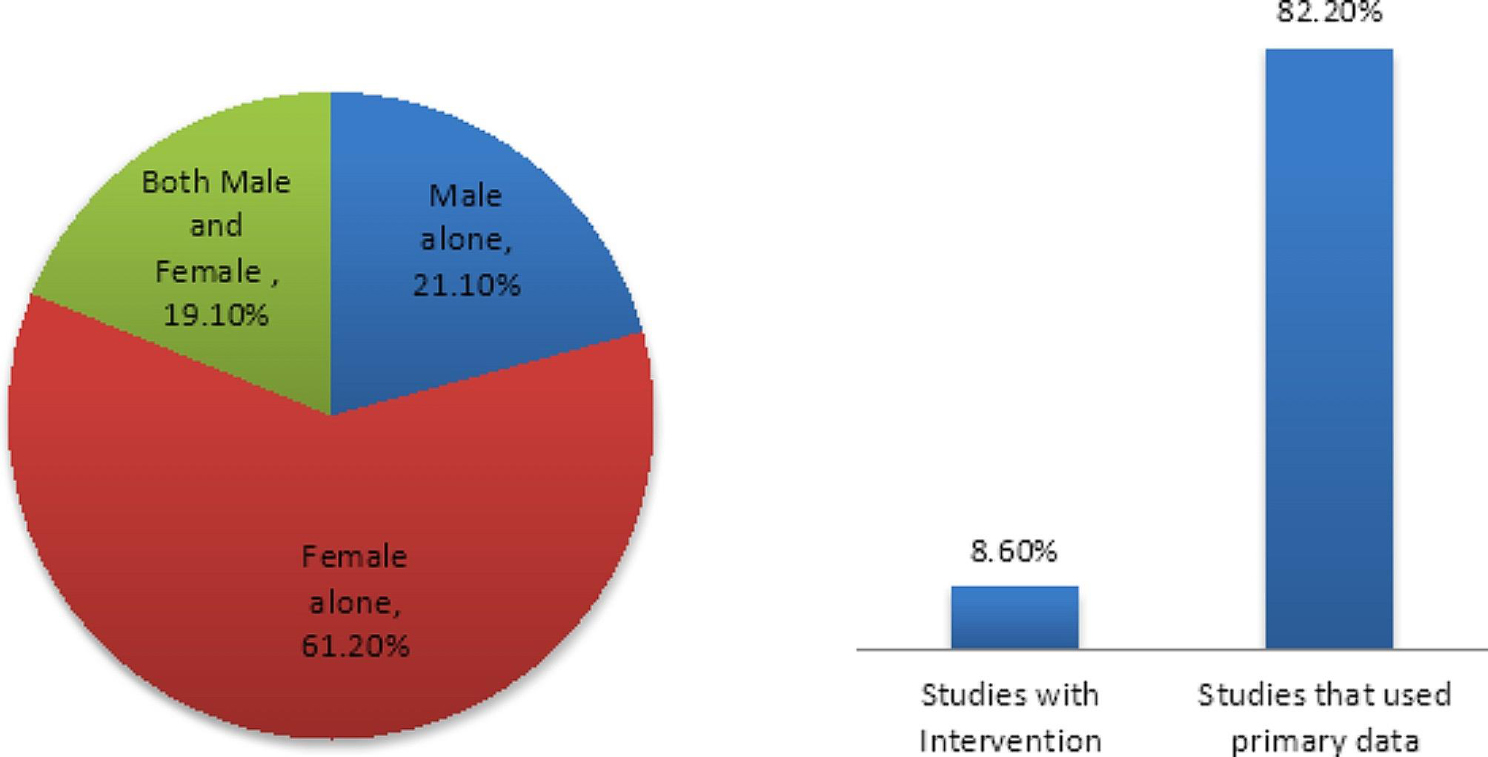
Methods used for reporting male involvement

As presented in Fig. 4, results show that 61.2% of studies on male involvement in FP collected data from women alone. Only about one-fifth (21.1%) of the studies obtained information from men, while 19.1% collected data from both men and women. The majority of the studies (82.2%) used primary data (data collected by the authors), while the rest used secondary data (e.g., Demographic and Health Survey Data).

As presented in Table 3, there are disparities in reporting male involvement in FP based on the reporting methods. While very few (7.5%) of studies in which responses were obtained from women measured male involvement as self-use, the most commonly adopted measure in studies that used men alone as respondents was self-use (75%). The most frequently adopted measure in studies that used women alone as respondents was asking them if they thought their partner supported FP (89.2%). The second most common method was asking women whether or not their partners (men) approved or consented to FP (52.7%). Expression of approval or consent for FP was the most commonly used measure of male involvement (69%) in studies that used both males and females as respondents. Less than half of the studies measured male involvement in FP by asking whether men/male participated or were engaged in discussion/communication about family planning. No more than 20% of the studies, regardless of who was used as respondents, measured male involvement through men’s attendance at family planning clinics.

**Table 3:** Disparities in reporting, based on reporting sources

	Measurement of Male Involvement in FP					
***Sources***	**Self-use (%)**	**FP Method choice (%)**	**Expressed approval or consent for FP (%)**	**Perceived support for FP**	**Discussion on family planning (%)**	**Accompany or attend a family planning clinic (%)**
Studies that obtained responses from men alone	75.0	46.9	46.9	0.0	25.0	18.8
Studies that obtained responses from women alone	7.53	19.4	52.7	89.2	45.2	16.1
Studies that obtained responses from both men and women	48.3	55.2	69.0	14.2	44.8	20.7

## Discussion

This Study was based on a bibliometric analysis of 152 peer-reviewed articles published between 1996 and 2023. The study identified and analyzed the various measures and concepts used to capture male involvement in FP in Africa. This analysis is crucial for understanding the validity of male involvement measures in FP and identifying the need for modification where required [30]. The study shows that the top three measures of male involvement in FP were expressed approval, inferred approval, and communication/discussion around FP. These measures are similar to some adopted by authors outside Africa [22, 23]. Within Africa, some of these metrics have also been used to measure male involvement in other sexual and reproductive health affairs, such as antenatal care, post-natal care, and child immunization [31, 32]. The predominant use of approval and communication as measures of male involvement in FP should not be surprising. This is because approval may suggest that men support FP and could motivate women to practice FP in a non-clandestine manner. Approval may create a healthy avenue for sexual partners to discuss FP and improve their knowledge of the benefits of FP to their peculiar situation [33]. When men approve of FP, it may legitimize women’s use of available resources, e.g., money to pay for services and time to visit the FP service center.

However, while men’s approval is essential [34], it may not translate to involvement in FP. According to the Cambridge Dictionary, ‘involvement’ connotes “the fact or condition of being involved with or participating in something.” It may thus be argued that measuring male involvement in FP through approval is not a valid measure of male involvement in FP. In many African settings where men are the breadwinners of a home/household/family [35, 36] and where FP costs are serviced from out-of-pocket payments [37], mere approval may not translate to women’s capacity to afford a suitable FP method. This lends credence to the role of women empowerment and reproductive agency in achieving FP goals [38] in view of some men’s ‘empty approval’ that may not translate to involvement.

Further, some of the reviewed studies also equated approval with the responsibility to pay for FP services. For instance, 12.4% of the studies measured male involvement in FP through men’s monetary provision for FP services and material costs. While men’s monetary provision may help access FP methods, especially where services are not free, relying on that may impose method choice (on women), which may not be preferred and thus impair the effectiveness. Available evidence shows that FP is most effective when couples use the most appropriate method recommended after a careful evaluation by competent providers [24, 39].

Our bibliometric analysis showed that about one-third of the analyzed studies measured male involvement in FP through men’s input when choosing the FP method. Fewer than this proportion measured male involvement through self-use, attendance at the FP clinic, deciding on an FP service center, and paying for FP services. Again, these measures of male involvement have been adopted in previous studies both within Africa [18, 19] and outside Africa [21]. One common feature of these measures is that they require action from men, unlike mere ‘approval’ that does not necessarily need men to act. Existing evidence shows that a known factor negatively affecting FP use is the fear or experience of adverse effects, which vary by FP methods [16, 40]. Therefore, where men are involved in the choice of FP method, this involvement may lead to the choice of a method with minimal adverse effects, which may improve their satisfaction with FP services. Men’s self-use of FP is arguably a valid measure of male involvement because it requires them to act.

However, men’s self-use may not be necessary if their female partners consistently and correctly use a modern method [19, 41]. This limits the applicability of men’s self-use for programmatic usage. It may be argued that attending or accompanying female partners to family planning clinics or service centers is one of the most encompassing measures of male involvement in FP. It captures men’s actions and signals their positive attitude to FP [41, 42]. Attending an FP clinic with female partners may reflect that both sexual partners discussed FP and agreed to explore its benefits. Moreover, through joint attendance, sexual partners would have the opportunity to get answers and clarity, which may positively shape their FP use experience. Furthermore, joint attendance implies genuine male support, empathy, and shared responsibility and may encourage women to use FP [34]. However, only 17.8% of the analyzed studies adopted this measure of male involvement in FP.

Furthermore, measuring male involvement through their input in deciding the choice of an FP service center is very important, but only 13.6% of the analyzed studies adopted this measure. In healthcare service delivery, where confidentiality is a significant determinant of health-seeking behavior [42, 43], men may be favorably disposed to accessing FP service in a service center of their choice, perhaps where confidentiality is guaranteed. This is more so because the gender norms in Africa tend to arrogate family planning roles to women [42, 44]. Under this norm, African men accompanying their wives to the FP clinic may be perceived as ‘less busy’ or ‘someone under control.’ While some African men may not be deterred by such socially undesirable labeling, most of them could be. Therefore, it is recommended that the measurement of male involvement in FP should incorporate men’s inputs in deciding the place of service delivery. Such input may be made by men when discussing FP with their partners, and this points to the centrality of communication between partners to achieve FP goals [45]. Where communication is lacking, misunderstanding may set in, and this could cause partners to act in secrecy, thus exposing them to reproductive health risks, e.g., unintended pregnancies.

Beyond the measurement and conceptualization of male involvement, our bibliometric analysis shows that more than three-fifths (61.2%) of the analyzed studies measured male involvement through responses obtained from women. This method is widely practiced in studies [18, 21], and the reason for this may not be far-fetched. One, it is a common assumption that women are more concerned than men about sexual and reproductive health matters and are more likely to report their FP situation [46, 47] accurately. Two, given men’s socially acceptable polygamous status in Africa [48], their involvement in FP may differ according to wives and may thus distort their reporting. Also, women are biologically configured to bear the burden of failed FP [49] disproportionately. These may explain why global goals, such as the Sustainable Development Goals (SDGs) 2030 and the Africa Agenda 2063, make a direct reference to females without commensurate references to males. While these points are valid, they should not suffice to mean that men’s side of the story need not be told during empirical investigations.

The non-incorporation of the male’s perspective and a preponderant use of non-valid measures of male involvement in FP may be why achieving target 3.7 of SDGs - universal access to family planning - does not appear to be in sight in Africa [50]. Not only have programs neglected men [51], research efforts seem not to have come to terms with the need to incorporate men’s perspectives into understanding how male involvement could be maximized. For instance, although the majority (82.2%) of the articles used primary data that permitted researchers to determine who their respondents would be, only 21.1% used information from males. Meanwhile, getting responses from men, or at least from both men and women, would have improved the quality of the reporting in some ways. Arguably, interviewing men about their involvement may create awareness that they are expected to be involved. It may provide a clearer understanding of what involvement means to men, which may help guide appropriate intervention to inform them about what their involvement should be. Also, since male FP methods exist [52, 53], including men as respondents in studies measuring male involvement could help unveil important information on promoting male method adoption.

An important takeaway from this study is that the many metrics used for measuring male involvement in FP make understanding what male involvement entails difficult. Given the complexities around gender issues and control dynamics, as recently seen in the context of adherence to HIV therapy [54], women who often require a male partner’s permission to access healthcare resources may perceive male involvement as a setback and could thus reject involving their partners in FP. Unfavorable male involvement could even be a clog in the wheel of women’s health and well-being in heterosexual relationships where women have little/no input in FP decision-making. Hence, we recommend two standard measures that entail men’s involvement and do not undermine women’s autonomy: (i) the proportion who engaged in ‘communication and discussion about FP with partners’ and (ii) The proportion of those who visited FP service centers with their partners within a reference period. In addition to the earlier stated advantages of adopting these measures, they show the level of intimacy amongst heterosexual partners. The activities that the measures depict allow couples to agree on FP goals mutually, thus facilitating their exercise of person-centered rights. Also, these measures would encourage the collection of union-specific data, which would not only be relevant in both polygamous and monogamous settings but would also improve accuracy for programmatic usage. When adopted, the standard measures of male involvement would make the evaluation of its impact clear and would thus provide evidence for its programmatic applications.

### Strength and limitations

This study documents the measures used to capture and conceptualize male involvement in FP in Africa. The study pointed out some metrics that could not validly measure male involvement but have been widely used in studies. Some measures that could validate male involvement but were largely neglected in studies were also identified. We suggested how these valid measures could be used with improvement, mainly through better men’s engagement in empirical investigations that focus on male involvement. An important strength of this study lies in its detailed description of the methods used to implement the study, thus promoting reproducibility - an essential tenet in scientific procedures. The study advances methodological adoption in social research by applying computational tools (Bibliometrix in R). However, the study has some limitations, which readers must be informed about. We did not include unpublished research articles and those published in journals not indexed by the searched databases (Scopus, Web of Science, and PubMed). This might explain why most of the reviewed studies had authors with affiliations in the United States (see Fig. 3), where there might be better access to funding support to cover the Article Processing Costs charged by journals indexed in these databases. However, while several publishers offer funding support to authors affiliated with African institutions to publish in journals indexed by the selected databases, we acknowledge that the choice of these databases might have led to the exclusion of some research articles written on male involvement in FP in Africa. We, however, justified such exclusion on the strength of preferring quality to quantity.

## Conclusion

Most studies have mainly measured male involvement in FP through expressed or perceived approval for FP, but these do not sufficiently capture male involvement and do not reflect women’s autonomy. Even though the reviewed studies claimed to have measured male involvement, very few obtained responses from men. We thus recommend measuring male involvement as the proportion who engaged in ‘communication and discussion about FP with their partners’ and the proportion who accompanied their partners to FP service centers. These measures would reflect the level of intimacy amongst heterosexual partners and encourage the collection of union-specific data. Also, a standard measure of male involvement in FP would make the evaluation of its impact clear and would provide evidence for its programmatic applications.

### Electronic supplementary material

Below is the link to the electronic supplementary material.

Supplementary Material 1

## Data Availability

The extracted data analysed in this study are available upon request. The corresponding author should be contacted for this.

## Abbreviations

FP: Family Planning
SDG: Sustainable Development Goals
