# Supplementary material for: Measurement and conceptualization of male involvement in family planning: a bibliometric analysis of Africa-based studies
Source: Contracept Reprod Med. 2024 Jun 13;9:29. doi: 10.1186/s40834-024-00293-9 (PMC11170783; doi:10.1186/s40834-024-00293-9)

**Appendix A** (string query)

( TITLE-ABS-KEY ( "male involvement" OR "male's involvement" OR "partner involvement" OR partner's involvement" OR "men involvement" OR "men involvement" OR "husband involvement" OR "husband involvement" OR "male participation" OR "male participation" OR "partner participation" OR "partner's participation" OR "men participation" OR "men participation" OR "husband participation" OR "husband participation") ) AND ( TITLE-ABS-KEY ( "contraceptive" OR "family planning" OR "contraception" ) ) AND ( LIMIT-TO ( PUBSTAGE , "final" ) ) AND ( LIMIT-TO ( LANGUAGE , "English" ) ) AND ( LIMIT-TO ( AFFILCOUNTRY , "Ethiopia" ) OR LIMIT-TO ( AFFILCOUNTRY , "Nigeria" ) OR LIMIT-TO ( AFFILCOUNTRY , "South Africa" ) OR LIMIT-TO ( AFFILCOUNTRY , "Uganda" ) OR LIMIT-TO ( AFFILCOUNTRY , "Kenya" ) OR LIMIT-TO ( AFFILCOUNTRY , "Ghana" ) OR LIMIT-TO ( AFFILCOUNTRY , "Tanzania" ) OR LIMIT-TO ( AFFILCOUNTRY , "Malawi" ) OR LIMIT-TO ( AFFILCOUNTRY , "Rwanda" ) OR LIMIT-TO ( AFFILCOUNTRY , "Mozambique" ) OR LIMIT-TO ( AFFILCOUNTRY , "Zimbabwe" ) OR LIMIT-TO ( AFFILCOUNTRY , "Senegal" ) OR LIMIT-TO ( AFFILCOUNTRY , "Zambia" ) OR LIMIT-TO ( AFFILCOUNTRY , "Cameroon" ) OR LIMIT-TO ( AFFILCOUNTRY , "Democratic Republic Congo" ) OR LIMIT-TO ( AFFILCOUNTRY , "Burkina Faso" ) OR LIMIT-TO ( AFFILCOUNTRY , "Egypt" ) OR LIMIT-TO ( AFFILCOUNTRY , "Sierra Leone" ) OR LIMIT-TO ( AFFILCOUNTRY , "Somalia" ) OR LIMIT-TO ( AFFILCOUNTRY , "Togo" ) OR LIMIT-TO ( AFFILCOUNTRY , "Angola" ) OR LIMIT-TO ( AFFILCOUNTRY , "Botswana" ) OR LIMIT-TO ( AFFILCOUNTRY , "Congo" ) )

**Appendix B** (co-concurrence network)


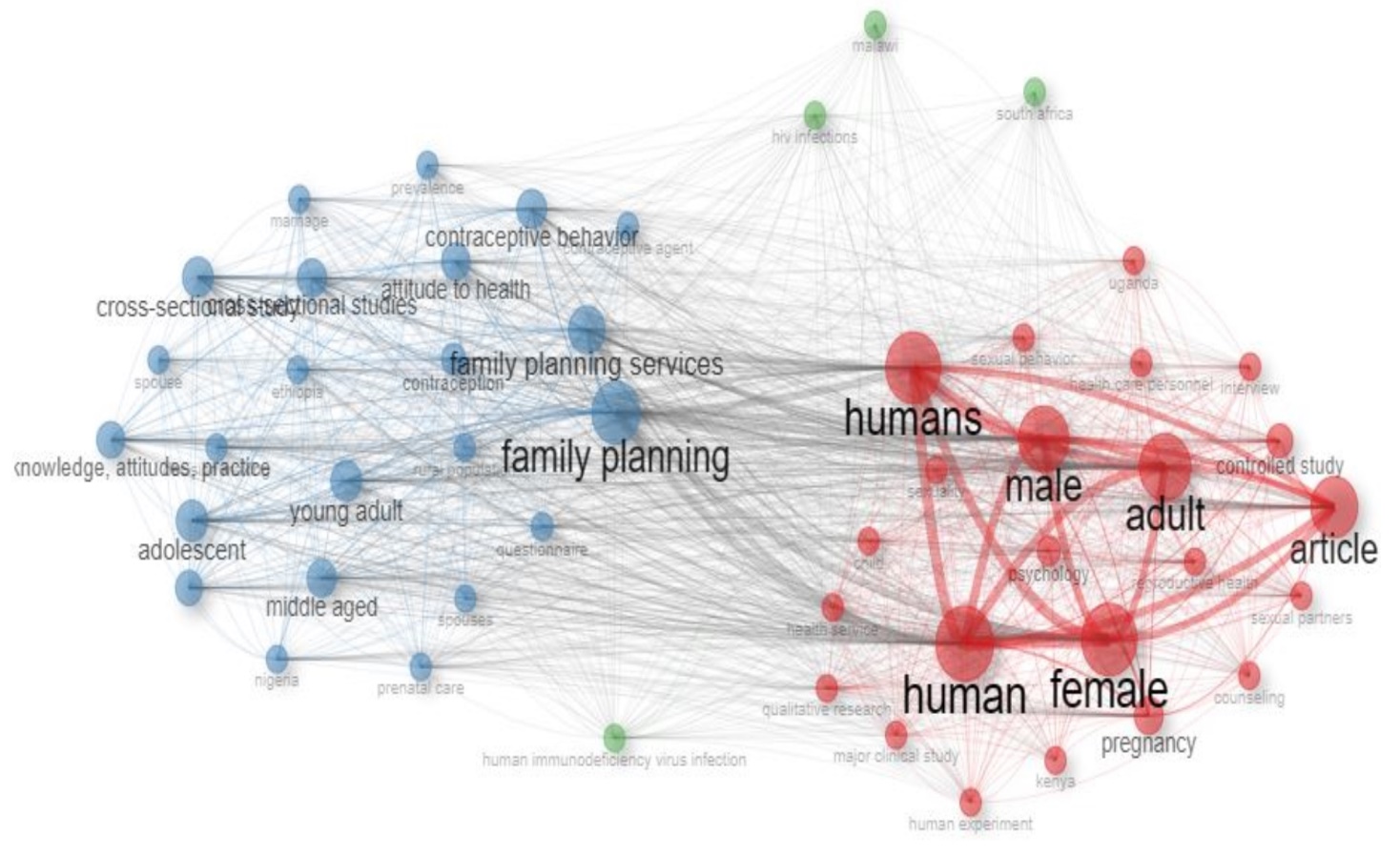

Supplement: Supplementary file 1 — Supplementary Material 1 [file 40834_2024_293_MOESM1_ESM.docx]
